# Imaging the adult zebrafish cone mosaic using optical coherence tomography

**DOI:** 10.1017/S0952523816000092

**Published:** 2016

**Authors:** ALISON L. HUCKENPAHLER, MELISSA A. WILK, ROBERT F. COOPER, FRANCIE MOEHRING, BRIAN A. LINK, JOSEPH CARROLL, ROSS F. COLLERY

**Affiliations:** 1 Department of Cell Biology, Neurobiology, and Anatomy, Medical College of Wisconsin, Milwaukee, Wisconsin; 2 Department of Psychology, University of Pennsylvania, Philadelphia, Pennsylvania; 3 Department of Ophthalmology, University of Pennsylvania, Philadelphia, Pennsylvania; 4 Department of Ophthalmology, Medical College of Wisconsin, Milwaukee, Wisconsin

**Keywords:** Optical coherence tomography, Zebrafish, Cone mosaic, Retina

## Abstract

Zebrafish (*Danio rerio*) provide many advantages as a model organism for studying ocular disease and development, and there is great interest in the ability to non-invasively assess their photoreceptor mosaic. Despite recent applications of scanning light ophthalmoscopy, fundus photography, and gonioscopy to *in vivo* imaging of the adult zebrafish eye, current techniques either lack accurate scaling information (limiting quantitative analyses) or require euthanizing the fish (precluding longitudinal analyses). Here we describe improved methods for imaging the adult zebrafish retina using spectral domain optical coherence tomography (OCT). Transgenic *fli1:eGFP* zebrafish were imaged using the Bioptigen Envisu R2200 broadband source OCT with a 12-mm telecentric probe to measure axial length and a mouse retina probe to acquire retinal volume scans subtending 1.2 × 1.2 mm nominally. *En face* summed volume projections were generated from the volume scans using custom software that allows the user to create contours tailored to specific retinal layer(s) of interest. Following imaging, the eyes were dissected for *ex vivo* fluorescence microscopy, and measurements of blood vessel branch points were compared to those made from the *en face* OCT images to determine the OCT lateral scale as a function of axial length. Using this scaling model, we imaged the photoreceptor layer of five wild-type zebrafish and quantified the density and packing geometry of the UV cone submosaic. Our *in vivo* cone density measurements agreed with measurements from previously published histology values. The method presented here allows accurate, quantitative assessment of cone structure *in vivo* and will be useful for longitudinal studies of the zebrafish cone mosaics.

## Introduction

Several characteristics of the zebrafish (*Danio rerio*) make it an ideal model for studying development and disease. The high homology between zebrafish and humans, along with the ease with which the zebrafish genome can be modified, allows for development and assessment of disease models that generally mimic human disease (Goldsmith & Jobin, 2012). In addition, zebrafish are easy to breed, mature quickly, produce large numbers of offspring, and are relatively inexpensive to maintain, making them an attractive option for many researchers (Pickart & Klee, 2014). Perhaps the most unique advantage to the zebrafish is its external development, which is highly conducive for studies of ocular development (Link & Collery, 2015). Of particular importance to studies of vision, the cone-rich retinas and diurnal vision of zebrafish complement the studies normally conducted in nocturnal, rod-dominant rodents (Allison et al., 2004; Chhetri et al., 2014).

Traditional confocal microscopy can be used with fluorescent markers and transgenic zebrafish lines to examine retinal structure and development both *ex vivo* (Li et al., 2012; Fraser et al., 2013; Raymond et al., 2014) and *in vivo* (Choi et al., 2010; Watanabe et al., 2010; Clark et al., 2011). *Ex vivo* studies of the zebrafish cone mosaic reveal a highly-organized tiling of four spectrally-distinct cone submosaics across the retina (Allison et al., 2010; Salbreux et al., 2012). Modification of a fluorescent stereomicroscope to include a custom gonioscopic lens has also enabled imaging of the cone mosaic *in vivo* (Duval et al., 2013), however, the images are of a relatively small retinal area and contain significant edge distortion. To cover a larger retinal area, several labs have adapted other ophthalmic imaging modalities for use in zebrafish. In one approach, Cheng et al. (2010) and Bell et al. (2014) used confocal scanning laser ophthalmoscopy to image the zebrafish retina *in vivo*. Fundus photography has also been used to image the optic disc, vasculature, and photoreceptors in the living zebrafish (Tschopp et al., 2010), though images of the photoreceptors with this method were not of high resolution. In addition, a major problem with many of these *in vivo* techniques is uncertainty regarding the lateral scale of the images, which limits the accuracy of any quantitative analysis.

Optical coherence tomography (OCT) permits non-invasive, high-resolution imaging of the eye in a variety of animal models (McLellan & Rasmussen, 2012). Compared to the previous techniques, OCT covers a wide field of view and has increased axial resolution (<5 microns). Recently, OCT has been applied to zebrafish to image the inner retina, lens, and anterior segment (Rao et al., 2006; Verma et al., 2007; Bailey et al., 2012; Weber et al., 2013; Collery et al., 2014). Given the interest in the photoreceptor layer, in particular its regenerative capacity (Wan & Goldman, 2016), we wanted to use OCT to image the zebrafish cone mosaic. The Bioptigen OCT built-in software uses a straight slab to generate *en face* projections, but due to the inherent curvature of the retina, the straight slab produces *en face* images that are not specific to a single layer of interest. In addition, since the probes used were designed for imaging the mouse retina, the lateral scale of images obtained in zebrafish is unknown (though recent work from Bailey et al. (2012) provided an OCT-histology correlation on the axial scaling of zebrafish OCT imagery). Here we sought to improve the method for generating *en face* projections, as well as determine the lateral scale of OCT images obtained in zebrafish. Using our methods, we demonstrate that quantitative data from OCT images of the photoreceptor mosaic agree with previously published histology data. Application of these methods will enable longitudinal, quantifiable imaging of zebrafish retinal structure in healthy and disease states.

## Materials and methods

To determine scaling of OCT images, axial length and retinal imaging were performed on seven *fli1:eGFP* zebrafish, which have fluorescently labeled vascular endothelial cells (Lawson & Weinstein, 2002). Zebrafish were anesthetized with 0.016% tricaine methanesulfonate, positioned on the imaging stage, and secured using a clay restraint (Collery et al., 2014). Axial length was determined using the optical path length measured from images obtained using the Bioptigen Envisu R2200 SD-OCT (Bioptigen, Research Triangle Park, NC) with a broadband source (central wavelength 878.4 nm, 186.3 nm bandwidth; Superlum, Enterprise Park, Cork, Ireland) and a 12-mm telecentric lens as previously described (Collery et al., 2014). High-resolution images of the retina were obtained using the mouse retina probe. Volume scans were nominally 1.2 × 1.2 mm with isotropic sampling (750 A scans/B scan; 750 B scans). Raw OCT scans for retinal images were exported and processed using a custom OCT volume viewer (Java software, Oracle Corporation, Redwood Shores, CA), in which an adjustable contour is used to generate *en face* summed volume projection (SVP) images (Flatter et al., 2014; Scoles et al., 2016). For a given B-scan, 3–15 control points were added to the initial contour, where each control point is manually adjusted to follow the contour of the layer(s) of interest. Contour thickness was adjusted to the maximum width of the retinal sublamina of interest, typically 10–20 pixels. The contour was manually adjusted for each B-scan in the volume, correcting for local changes in layer topography and gross changes in axial position due to breathing artifacts. The OCT volume viewing code is available on request. Multiple *en face* images can be generated for each OCT volume, resulting in images of different retinal features (*e.g*., inner retinal vasculature and photoreceptor mosaic).

Following OCT imaging, the *fli1:eGFP* fish were terminally anesthetized and decapitated, and heads were fixed in 4% formaldehyde overnight. The eyes were enucleated and the anterior segment removed from the eyecup. The eyecups were imaged with a Nikon Eclipse E600FN confocal fluorescent microscope with a Nikon D-eclipse C1 camera attachment (Nikon, Tokyo) to view the fluorescently labeled vasculature of the retina. Of the 14 eyes imaged, nine eyes (from seven fish) had minimal post-mortem distortion and were included for subsequent analysis. From microscopy images, the distance between blood vessel branch points was measured in microns using Nikon's EZ-C1 3.90 Free Viewer, with a minimum of three measurements per eye. Accuracy of the fluorescent microscopy measurements was confirmed by imaging a calibration slide at the same magnification. Identical blood vessel branch point measurements were made on the *en face* OCT images using ImageJ (Schneider et al., 2012). The ratio of *ex vivo* (microns) to *in vivo* (pixels) distance was calculated for each corresponding measurement and averaged for each eye. This micron/pixel ratio was used to calculate the actual scan width by multiplying it by the number of A scans/B scan (the scan width in pixels). The ratio of the actual scan width to the nominal scan width (calculated based on the known scan angle and assumed mouse optics) was then plotted against axial length to determine lateral scaling of OCT images as a function of eye length.

An additional five wild type zebrafish were imaged following the protocol above to gather data on the peripheral photoreceptor mosaic, which was defined as 20–50 microns from the optic nerve and corresponded to the transition from disorganized embryonic growth to crystalline adult growth. *En face* images were generated using the OCT volume viewer (Flatter et al., 2014; Scoles et al., 2016). Contours were positioned at the most anterior photoreceptor layer to generate *en face* images of the UV cone layer and at the most posterior photoreceptor layer to generate *en face* images of the red/green cone layer (Branchek & Bremiller, 1984). Cones were identified using a semi-automated algorithm with manual correction (Garrioch et al., 2012). Density and mosaic geometry were assessed from the resultant cone coordinates using a custom program as previously described (Cooper et al., 2016).

## Results

**Fig. 1.**
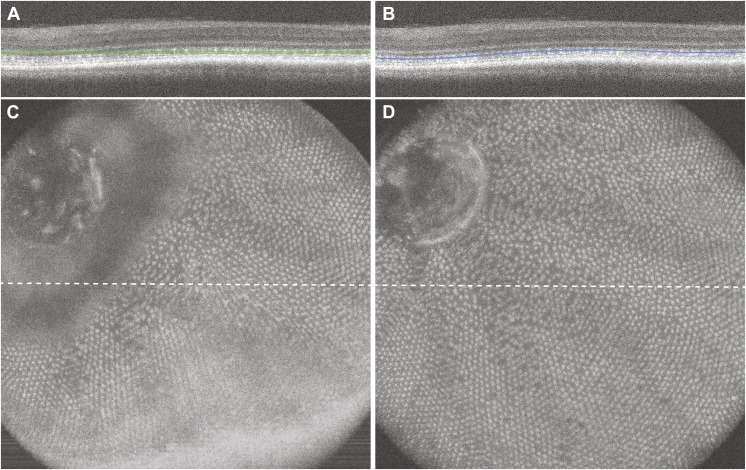
Visualization of the zebrafish cone mosaic using *en face* volume projection images. (**A**, **C**) The straight slab method in the native Bioptigen analysis software results in an *en face* volume projection with different retinal layers included at different locations within the volume, thus the cone mosaic is only visible in some parts of the *en face* image. (**B**, **D**) Using a custom contour that follows a single outer hyper-reflective band results in an *en face* projection of that layer, in this case the UV cone mosaic.

Using the custom volume viewer, distinct sublaminas are easily visualized and are uniform across the image. Fig. 1 illustrates representative SVP image quality that can be obtained using the custom OCT volume viewer compared to the built-in software. By creating a custom contour (Fig. 1B), we can generate SVP images of a single retinal layer (UV cone layer shown in Fig. 1D), compared to the corresponding image generated from a flat slab as done with the built-in Bioptigen software (Fig. 1A and 1C). The custom software generates significantly improved images compared to commercially available software.

**Fig. 2.**
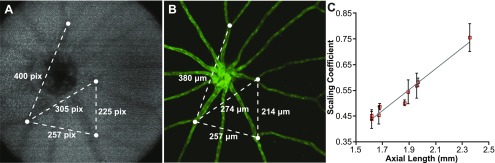
Deriving the lateral scale of *in vivo* OCT images of the *fli1:eGFP* zebrafish retina. (**A**) *En face* image generated by positioning the custom contour within the retinal nerve fiber layer. Measurements (in pixels) were taken between multiple blood vessel branch points (white dots). (**B**) Corresponding *ex vivo* fluorescent microscopy image of the same retina, with measurements (in *µ*m) taken between the same blood vessel branch points in (**A**). The OCT:microscopy measurements were averaged for each eye and used to determine the size of the OCT scan in *µ*m. A scaling coefficient for each scan was calculated as the ratio between the actual size of the OCT scan to the nominal OCT scan size (in this case, 1200 *µ*m). (**C**) The scaling coefficient for each scan was plotted against the axial length for that eye and fit with a linear model. Error bars represent one standard deviation for each eye.

Using *en face* images generated at the level above the nerve fiber layer, we were able to identify distinct branch points in the retinal blood vessels (Fig. 2A). The corresponding *ex vivo* measurements are shown in Fig. 2B. The ratio of the actual scan width to the nominal scan width (scaling coefficient) was plotted as a function of axial length (Fig. 2C). There is a significant positive correlation between the scaling coefficient and axial length (*r* = 0.98, *P* < 0.0001; Pearson's correlation), such that as axial length increases, the actual retinal area covered by the scan increases. A linear fit to these data provides a mathematical formula for calculating the scaling coefficient (s) for any zebrafish OCT based on axial length. This can be calculated as follows:1
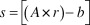
where *A* is the axial length in millimeters, and *b* and *r* are fixed values 0.2187 and 0.4059, respectively. It should be noted that this relationship has been adjusted to account for differences in scan settings and is therefore independent of the field of view (FOV), number of A scans/B scan, or number of B scans used.

**Fig. 3.**
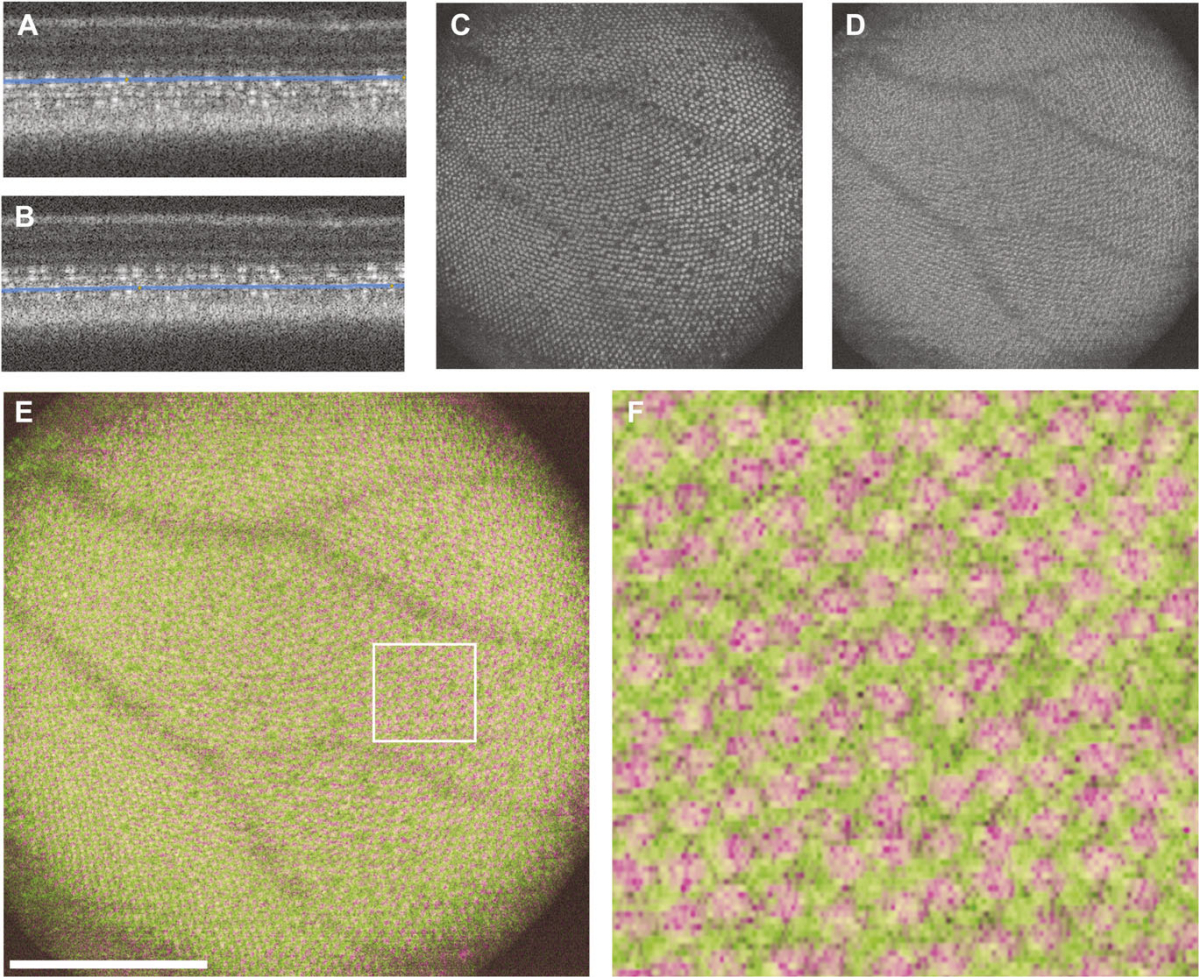
Visualization of the UV and red/green photoreceptor layers. (**A**) B scan with contour placed at the UV layer and (**B**) the same B scan with the contour moved to the red/green layer. The resultant images generated produce *en face* images of (**C**) the UV cone layer and (**D**) the red/green cone layer. A false-colored overlay (**E**) of these two layers showing the alternating/interleaved geometry of these photoreceptor sublamina. Scale bar = 200 *µ*m. (**F**) A cropped region shows the interleaved red/green and UV cone mosaics.

**Fig. 4.**
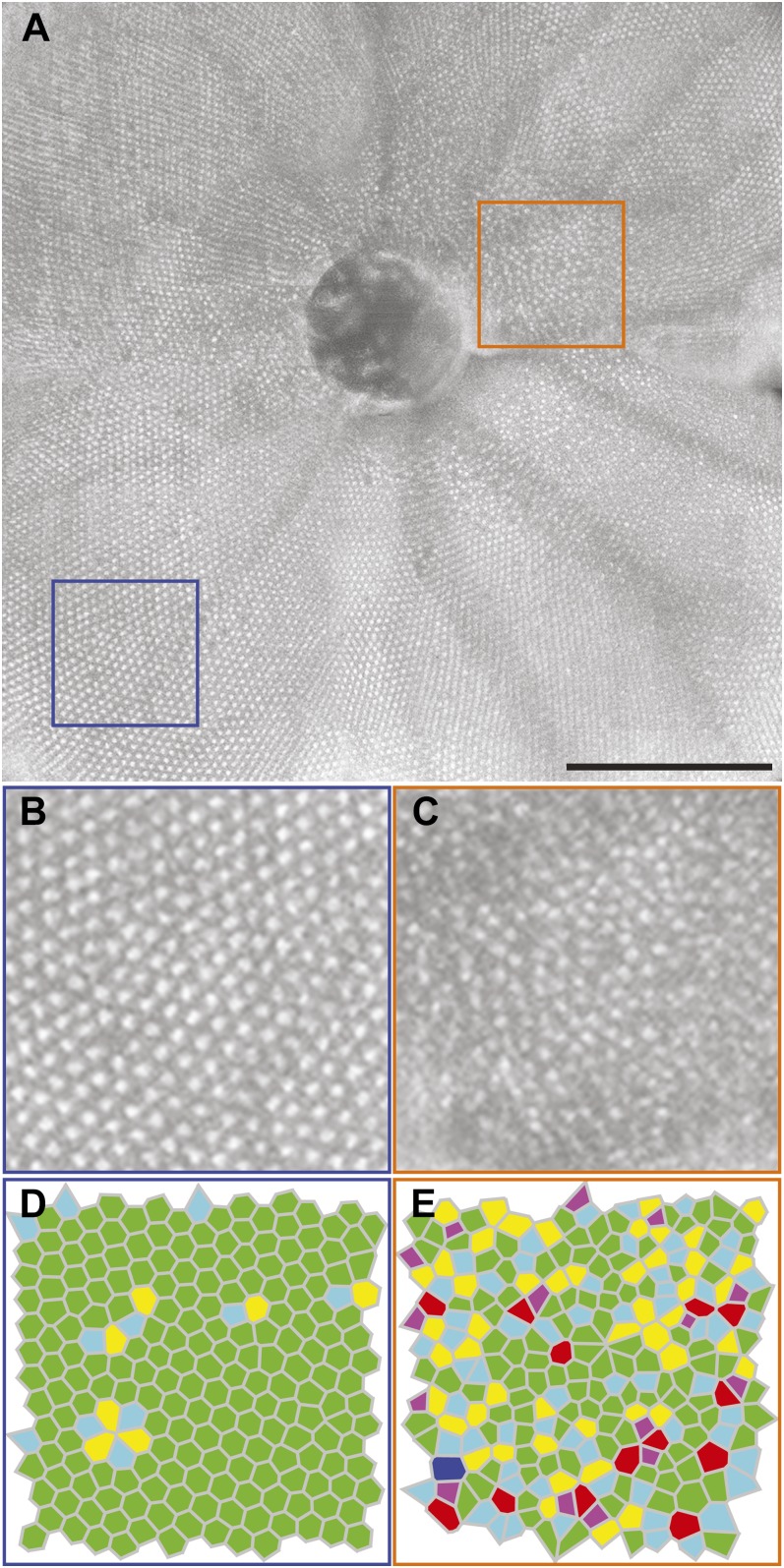
Regional differences in the topography of the zebrafish cone mosaic. (**A**) Montage of five *en face* images of the adult zebrafish UV cone sublamina. Scale bar = 200 *µ*m. Cropped images of the (**B**) peripheral adult growth and (**C**) parapapillary larval growth, showing differences in the percentage of six-sided cells within the cone mosaic. The corresponding Voronoi diagrams indicate the number of sides for each Voronoi cell (purple = 4; light blue = 5; green = 6; yellow = 7; red = 8; dark blue = 9). (**D**) The peripheral adult growth (blue box in **A**) shows a regular, crystalline mosaic, indicated by the high number of six-sided Voronoi cells (green cells). (**E**) In contrast, the parapapillary larval growth (orange box in **A**) shows disorganized, random packing.

Wild-type zebrafish were imaged and analyzed as described above for the *fli1-eGFP* fish. Using the scaling formula [eqn (1)] and the axial length, we determined the size of the OCT scan for each eye. The *en face* images of the cone mosaic produced with this method show similar packing arrangements and photoreceptor tiering that have been previously observed *ex vivo* (Allison et al., 2010; Tarboush et al., 2012; Ramsey & Perkins, 2013). Contours generated at the innermost photoreceptor layer (corresponding to the UV photoreceptor layer) (Fig. 3A) and the outermost photoreceptor layer (corresponding to the red/green photoreceptor layer) (Fig. 3B), produce *en face* images with non-overlapping cone structures (Fig. 3C and 3D). A false color overlay (Fig. 3E and 3F) shows that these separate photoreceptor layers are interleaved with one another as seen in histology (Salbreux et al., 2012). We are able to observe the disorganized packing near the optic nerve, made up of cones that formed during larval development (Fig. 4C and 4E), and the crystalline organization of the cones that developed as the fish transitioned to adulthood (Fig. 4B and 4D). Analysis of the peripheral UV cone sublamina shows a highly crystalline photoreceptor mosaic with 67.6 ± 11.0% of UV cones displaying 6-sided geometry. Within the images analyzed, we observed regions of even greater regularity, with some patches of the mosaic having 96% six-sided Voronoi cells; these patches were as large as 0.09 mm^2^. Average UV cone density was found to be 19,557 ± 4,716 cones/mm^2^, with values ranging from 12,593 to 25,756 cones/mm^2^. These values agree with previously published wild-type histology values ranging from about 6,000 to 23,000 cones/mm^2^ (Allison et al., 2010; Salbreux et al., 2012; Duval et al., 2013; Raymond et al., 2014).

## Discussion

Here we present a method for deriving accurate, quantitative measures of the zebrafish cone mosaic. The main improvements of our approach include utilizing a custom contour method to generate *en face* images from the OCT volumes as well as deriving accurate estimates of the lateral scale of the *in vivo* images acquired using this system. Individual differences in axial length (which affect optical magnification) have been shown to affect the accuracy of OCT measurements in the human retina (Odell et al., 2011; Parthasarathy & Bhende, 2015). In animals, correction of the lateral magnification of OCT images in the rat eye has been done by developing a schematic model eye (Lozano & Twa, 2013), and correction of the axial scale of OCT images in the zebrafish eye has been done using correlative histology (Bailey et al., 2012); however, to our knowledge, lateral scaling has not previously been determined for zebrafish. It should be noted that our method applies only to this specific OCT system (Bioptigen OCT with mouse probe), and a similar one-time calibration would be needed to determine scaling for different OCT systems. As a result of our methodology, we were able to make quantitative *in vivo* measurements of the adult zebrafish cone mosaic.

The current study had some limitations. First, analysis of the cone mosaic was limited to the UV sublamina, which has larger cones that are spaced farther apart than the other sublamina (Robinson et al., 1993). Although the red/green (L/M) cone sublamina can be imaged using this method (Huckenpahler et al., 2016), the small size and compact spacing are sometimes below the resolution of the OCT. Similarly, the blue (S) cone sublamina is infrequently distinguished with our current system. System improvements such as using a bore designed specifically for the zebrafish eye, or employing adaptive optics OCT (AO-OCT) for imaging (Jian et al., 2013; Levine et al., 2013) could improve the resolution. A second limitation is that the creation of custom contours for *en face* images is a time-consuming process, as the contours are adjusted for each B scan within the volume. Combining our *en face* method with automated segmentation algorithms (Garvin et al., 2009; Wang et al., 2012; Chiu et al., 2013) could significantly improve processing time. A final limitation is the inability to combine fluorescent labeling with this imaging approach, though molecular labels that manipulate light scattering, such as gold nanoparticles, have been successfully used with OCT (Hayashi et al., 2009; de la Zerda et al., 2015). Such tools may improve the ability to label specific cell populations within the OCT volumes (and thus the *en face* images).

Despite these limitations, OCT permits non-invasive, high resolution imaging of the eye in a variety of animal models (McLellan & Rasmussen, 2012). Although OCT has been largely relegated to measuring retinal thickness, the ability to generate accurately scaled *en face* images increases the application of this method for structural and functional studies. In humans, OCT *en face* images are used to identify and track retinal lesions and to monitor disease progression (Sallo et al., 2012; Ferrara et al., 2014; Flatter et al., 2014; Mohammad et al., 2014; Hood et al., 2015). Studies in frogs and mice have applied *en face* imaging to study phototropic changes and dysfunction in photoreceptors (Zhang et al., 2012; Lu et al., 2013) and *ex vivo en face* imaging has been used to study photoreceptor development in zebrafish (Mitchell et al., 2015). The *in vivo* nature of the method described here permits longitudinal assessment of the mosaic in the same animal over time and we foresee this technique having wide application to developmental biology, drug discovery, and disease modeling. We anticipate that researchers would likely use this technique to study longitudinal changes to the cone mosaic in normal development, or in response to disease. However, given the popularity of the zebrafish for drug screening, we also envision a more clinical application where OCT imaging is used to monitor drug oculotoxicity or efficacy prior to FDA approval. Moving forward, applying these imaging methods and robust analysis tools in a variety of disease models could significantly improve our understanding of retinal structure and disease.
